# Progressive Mechanical and Structural Changes in Anterior Cerebral Arteries with Alzheimer’s Disease

**DOI:** 10.21203/rs.3.rs-3283587/v1

**Published:** 2023-08-29

**Authors:** Xiaozhu Liu, Samuel Halvorsen, Nathan Blanke, Margaret Downs, Thor D. Stein, Irving J. Bigio, Joseph Zaia, Yanhang Zhang

**Affiliations:** Boston University; Boston University; Boston University; Boston University Chobanian & Avedisian School of Medicine; Boston University; Boston University; Boston University Chobanian & Avedisian School of Medicine; Boston University

**Keywords:** Alzheimer’s disease, anterior cerebral artery, cerebrovascular remodeling, smooth muscle cell atrophy, elastin degradation, collagen

## Abstract

Alzheimer disease (AD) is a neurodegenerative disease and the main cause for dementia. The irreversible neurodegeneration leads to a gradual loss of brain function characterized predominantly by memory loss. Cerebrovascular changes are common neuropathologic findings in aged subjects with dementia. Cerebrovascular integrity is critical for proper metabolism and perfusion of the brain, as cerebrovascular remodeling may render the brain more susceptible to pulse pressure and may be associated with poorer cognitive performance and greater risk of cerebrovascular events. The objective of this study is to provide understanding of cerebrovascular remodeling with AD progression. A total of 28 brain donor participants with human anterior cerebral artery (ACA) from controls and pathologically diagnosed AD groups (early – Braak stages I-II; intermediate – Braak stages III-IV; and advanced – Braak stages V-VI) were included in this study. Mechanical testing, histology, advanced optical imaging, and mass spectrometry were performed to study the progressive structural and functional changes of ACAs with AD progression. Biaxial extension-inflation tests showed that ACAs became progressively less compliant, and the longitudinal stress in the intermediate& advanced AD groups was significantly higher than that from the control group. With pathological AD development, the inner and outer diameter of ACA remained almost unchanged; however, histology study revealed progressive smooth muscle cell atrophy and loss of elastic fibers which led to compromised structural integrity of the arterial wall. Multiphoton imaging demonstrated elastin degradation at the media-adventitia interface, which led to the formation of an empty band of 21.0 ± 15.4 μm and 32.8 ± 9.24 μm in width for the intermediate& advanced AD groups, respectively. Furthermore, quantitative birefringence microscopy showed disorganized adventitial collagen with AD development. Mass spectrometry analysis provided further evidence of altered collagen content and other extracellular matrix (ECM) molecule and smooth muscle cell changes that were consistent with the mechanical and structural alterations. Collectively, our study provides understanding of the mechanical and structural cerebrovascular deterioration in cerebral arteries with AD, which may be related to neurodegenration and pathology in the brain.

## Introduction

Alzheimer’s Disease (AD), a neurodegenerative disease characterized by the appearance of neurofibrillary tangles and accumulation of amyloid-β (Aβ) peptide in brain (Duyckaerts et al., 2009; Jellinger, 2002), is the most prevalent cause of dementia (Alzheimer’s Association, 2019). As AD progresses, brain atrophy occurs and patients lose brain functions corresponding to the atrophy location, which leads to gradual cognitive decline, personality changes, and eventual inability to sustain their activities of daily living independently (Shin et al., 2005). Cerebrovascular changes are common neuropathologic findings in aged subjects with dementia (Marlatt et al., 2008). With neurodegeneration in dementias such as AD, neurovascular coupling is impaired and results in perfusion dysfunction, which renders the brain more susceptible to high pressure (Mancia & Grassi, 2004).

Despite the close association between nerves and vasculature, compared with studies on neurons, cerebrovascular remodeling during the progression of neurological disorders and the neurovascular relationships in the brain are less delineated. Among the limited studies, using combined clinical diagnosis and noninvasive assessments such as MRI or transcranial Doppler ultrasonography, cerebrovascular alterations in AD were reported. An increase in arterial stiffness was found in patients with AD compared to the control group (Rivera-Rivera et al., 2021), and there was a strong association between compliance reduction in cerebral arteries and presumptive AD diagnosis (Roher et al., 2011). However, a direct assessment relating the biomechanical behavior of cerebral arteries with AD progression is lacking.

Compliance of large cerebral arteries is critical as these arteries dampen the pulsatile pressure and protect the microcirculation and blood brain barrier (BBB) from damage (Hughes et al., 2015). Cerebrovascular dysfunction can render the brain more susceptible to pulse pressure and lead to BBB breakdown (Kisler et al., 2017). Aβ normally can be eliminated from BBB along blood vessels via artery contractions (Arbel-Ornath et al., 2013; Cockerill et al., 2018). With impaired BBB and vascular contractibility, Aβ is more likely to deposit in brain tissues and the cerebrovascular system (Austin et al., 2011). Studies showed that less compliant cerebral arteries lead to ineffective clearance of Aβ and other toxic metabolites (Govindpani et al., 2019; ladecola & Gottesman, 2018). Additionaly, Aβ deposition in the arterial wall was shown to be a major cause of microhemorrage in AD (Itoh et al., 1993). Therfore, compliance of large cerebrovascular arteries plays a critcal role in the clearance of Aβ and other toxic metabolites in the brain. Aβ deposition, due to loss of compliance of large cerebral arteries, results in smooth muscle cells (SMC) atrophy, causing further decrease of vascular contractability and cerebral blood flow (Hald et al., 2016; Thal et al., 2008), thus representing a vicious cycle.

Cerebrovascular dysfunction can have detrimental impacts on the brain and is closely associated with cognitive impairment. The goal of this study is to provide an understanding of cerebrovascular remodeling in the progression of AD. Human anterior cerebral artery (ACA), which originates in the Circle of Willis and supplies blood to the upper and medial cerebrum surfaces, was used in this study. To establish an understanding of the changes in mechanical properties of cerebrovascular arteries with AD progression, biaxial extension-inflation tests were performed on ACAs from the control and pathologically diagnosed AD groups, from which the stress vs. stretch relationships in circumferential and longitudinal directions were obtained. Additionally, histology studies, multiphoton imaging, birefringence microscopy, and mass spectrometry were performed to examine structural integrity of the arterial wall. These studies showed progressive media atrophy, elastic fiber degradation, and adventitia structural disorganization in the cerebrovascular tissue that correlated with AD development.

## Material and Methods

### Sample preparation

Human ACAs from brain donors with neuropathological assessments were obtained from the NIH NeuroBioBank. Gross and microscopic neuropathology including assessments of neuritic beta-amyloid plaque density and Braak and Braak stage for neurofibrillary tangles were provided. A total of 28 brain donor participants were examined and included controls without neuritic beta-amyloid plaques (n = 8) as well as 20 participants with AD pathology defined by moderate to severe neuritic beta-amyloid plaque density that were further stratified by Braak and Braak stage to include Braak stages I and II (n = 6, early stage), Braak stages III and IV (n = 4, intermediate stage), and Braak stages V and VI (n = 10, advanced stage) in Table 1.

Samples were kept frozen in dry ice during transportation and were then stored in a −80°C refrigerator in lab. Before experiments arteries were placed in a −20°C freezer overnight and then defrosted at room temperature before experiments. Blood in the arteries was rinsed out using 1× phosphate buffered saline (PBS). Samples usually arrived with the left and right ACA branches connected by the anterior communicating artery. The pre- and post-communicating segment of ACAs are referred as the first and second ACA segment. The second segment of the ACA was carefully dissected, and connective tissue was removed. To obtain more consistent results of arterial integrity changes with AD and corresponding mechanical behavior, all tested ACA segments were chosen from areas without visible atherosclerosis. Small side branches on the ACAs were tied off with 7 – 0 nylon sutures before mechanical testing.

### Biomechanical characterization and data analysis

Biaxial extension-inflation tests were performed using a pressure myograph (110P XL, DMT Inc., Denmark) which measures the axial force, transmural pressure, and arterial outer diameter during testing. The ACAs were submerged in 37°C PBS, mounted on stainless steel cannulas, and then secured with 6 – 0 nylon sutures at both ends. The *in vivo* stretch ratio was determined when the axial force variation was minimized during pressure loading (Ferruzzi et a., 2013). For preconditioning, the ACAs were inflated from 0 to 40 mmHg, 0 to 60 mmHg, and finally 0 to 80 mm Hg at the *in vivo* stretch ratios (Wagner & Humphrey, 2011). For biaxal extension-inflation test, the ACAs were loaded at the *in vivo* stretch ratio to 80 mm Hg, the average mean arterial pressure of human ACA (Rosenberg et al., 1985), and then unloaded to zero pressure with an increment of 10 mmHg at the speed of 1.5 mmHg/sec.

The extension-inflation test was repeated 3 times at the *in vivo* stretch ratio to improve the reliability and repeatability, and the last set of data was used for later analysis (Ferruzzi et al., 2013). After the test, two 1-mm rings were cut off from both ends of the arteries to measure the average reference artery dimensions. The outer and inner perimeter of the rings were measured using an image processing tool, FIJI (http://fiji.sc/Fiji, Ashburn, VA) with a segmented line tool to calculate the undeformed outer radius $R_{o}$ and the inner radius $R_{i}$. The initial artery length L was measured between sutures before the test and the stretched artery length l was recorded in biaxial extension-inflation test to calculate the axial stretch ratio, $\lambda_{z}=\frac{l}{L}$. Assuming incompressibility (Humphrey 2013), the deformed inner radius, $r_{i}$, was obtained as:

1 $$r_{i}=\sqrt{r_{o}^{2}-\frac{R_{o}^{2}\;-\;R_{i}^{2}}{\lambda_{z}}}$$

where ro is the deformed outer radius. The stretch in the circumferential direction was then calculated:

(2) $$\lambda_{\vartheta }=\frac{r_{o}+r_{i}}{R_{o}+R_{i}}$$

The circumferential and axial stresses within the arterial wall were calculated as:

(3) $$\sigma_{\vartheta }=\frac{Pr_{i}}{r_{o}-r_{i}}\;\text{and}\;\sigma_{z}=\frac{f_{T}+P\pi r_{i}^{2}}{\pi \left(r_{o}-r_{i}\right)\left(r_{i}+r_{o}\right)}$$

where $f_{T}$ represents the axial force, and the P is the transmural pressure. Circumferential stretch was normalized via dividing by the circumferential stretch at 0 mmHg in the same loading cycle. Circumferential stress and circumferential stretch at 80 mmHg were compared between different pathology groups. The circumferential stress versus circumferential stretch curve was fitted with an exponential function, and the tangent stiffness was then derived for further comparison.

### Histology imaging

To reveal structural changes in the arterial wall with AD development, Movat’s stain was used which stains elastic fibers in black, collagen fibers in yellow, and smooth muscles in red color. Briefly, samples from age-matched control and AD groups were fixed in 4% paraformaldehyde overnight, embedded in paraffin, cut into 5 μm slices and then stained. Stained slides were viewed and scanned using an Olympus VS120 automated slide scanner. Unstained slides were also prepared for birefringent microscopy using the same protocol without staining. For birefringent imaging, paraffin was removed from slides using xylene (Korbler et al., 2003; Westermark et al., 1999).

### Multiphoton imaging

Multiphoton images of artery samples were acquired with a multiphoton microscope (Carl Zeiss LSM 710 NLO) using a 20× water immersion objective lens. Here samples were obtained from age-matched male ACAs from the control and AD groups that were used for histology. Rings about 0.5 mm in length were cut from ACAs after biomechanical testing and submerged in 1× PBS to view the cross-section of the arterial wall. The femtosecond IR Pulse laser was set to 810 nm to generate two-photon excited fluorescence (2PEF) of elastin (525/45 nm) and second harmonic generation (SHG) of collagen (417/80 nm) (Chow et al. 2014; Yu et al. 2018a, b). Z-stack images of the arterial cross-section were acquired ranging from 70–150 μm in depth with 1 μm spacing between adjacent images. Images with a field of view of 425 μm × 425 μm were obtained to reveal the cross-sectional view of elastic and collagen fiber distributions in the ACA.

To determine if an empty band within the elastic fiber network forms at the media-adventitia interface during AD progression, signal intensity was measured using grayscale maximum intensity projection images of elastin using FIJI. Three samples were selected from each AD stage, and for each sample the empty band distance was obtained by averaging the band width measured at four different locations in the cross section. Images were rotated to vertically align the arterial wall and thresholded to remove noise. The binarized image was discretized into a 30×30 grid and a rectangular region of interest was selected to measure signal intensity. The area fraction occupied by black pixels was determined for each square region, and an average area fraction in each column was calculated and used to determine the existence of an empty band in the elastic fiber network at the interface of media and adventitia. The empty band was defined for columns with average area fraction values two standard deviations lower than that of the of the media and adventitia.

### Birefringence microscopy

Quantitative birefringence microscopy (qBRM) (Blanke et al., 2021) was used to perform characterization of the ACA wall structure by imaging their structural birefringence. For label-free qBRM of the ACAs, unstained sections were imaged with a narrowband red LED source and either cross-circular-polarized birefringence microscopy (CCP-BRM), consisting of circular polarizers of opposite handedness in the illumination and detection arms, or with qBRM, consisting of a rotating linear polarizer in the illumination arm and a circular analyzer in the detection arm (Glazer et al., 1996). CCP-BRM was used to acquire full-sample images, taken with a 20x objective (Olympus UPLFLN20XP), which were stitched together using the BaSiC plugin (Peng et al., 2017) in Fiji. After acquiring full-sample images, a horizontally aligned region of the arterial wall was selected and imaged with qBRM using the same 20× objective. During qBRM, a set of six images was taken during stepwise rotation of the linear polarizer (at 30-degree increments). In the resulting image sets, the intensity variation of each pixel can be analyzed following the steps detailed in (Blanke et al., 2021), providing quantitative birefringence parameter maps of relative retardance and in-plane optic-axis orientation. In the qBRM images, the relative retardance corresponds to the density and degree of alignment, while the in-plane optic-axis orientation map corresponds to the direction of anisotropy of the medium. The relative retardance map is displayed as a grayscale intensity image and is used to “weight” the optic-axis orientation maps for visualization. In the retardance-weighted optic-axis orientation maps, the direction of anisotropy for each pixel is displayed based on a color-coded orientation wheel and the relative retardance provides the intensity of the image.

### Mass spectrometry

Proteomics was conducted on ACAs from the control (n = 5) and advanced AD (n = 6) groups using on-slide digestion to extract glycosaminoglycans and proteins (Raghunathan et al., 2019). Briefly, chondroitinase ABC, heparin lyases I, II, and III, and trypsin/Lys-C were applied to the surface of 1 mm artery rings and the digested glycosaminoglycans and peptides were extracted. Peptides were cleaned using C-18 spin columns and then analyzed using a nanoAcquity UPLC (Waters Technology Corp.) interfaced with a Q-Exactive HF mass spectrometer (ThermoFisher Scientific). Reversed-phase C-18 analytical (BEH C18, 150 μm × 100 mm) and trapping (180 μm × 20 mm) columns from Waters technology were used with a 75 min LC gradient from 2 to 98% acetonitrile in 55 min, using 99% water/1% acetonitrile/0.1% formic acid as mobile phase A and 99% acetonitrile/1% water/0.1% formic acid as mobile phase B at a flow rate of 0.5 μl/min. Data-dependent tandem MS was acquired in the positive ionization mode for the top 20 most abundant precursor ions. Full MS scans were acquired from *m/z* 350 to 2,000 with 60,000 resolution using an automatic gain control target of 3×10^6^ and maximum injection time (IT) of 100 ms. Dynamic exclusion (10 s) was enabled. Precursor ions were fragmented using a resolution of 15,000 with a maximum injection time of 50 ms and an automatic gain control value of 2e5 using higher energy collision-induced dissociation with a stepped normalized collision energy of 27 and 35 V. Proteomics database search and label-free quantitation were performed using PEAKS X+ (Bioinformatics Solutions, Inc.) to obtain a list of proteins present in the specimens and determine which proteins were differentially expressed.

### Statistical analysis

Student t-tests and one-way ANOVA were performed on *in vivo* stretch ratio, undeformed arterial dimensions including thickness and outer and inner diameter, tangent stiffness obtained from the circumferential stress-stretch curves, and the width of empty band region in the elastic fiber network from the control and AD groups (*p< 0.05). Results are presented as mean ± standard error of the mean.

For proteomics data, protein and protein-peptide lists (Figs. S1 and S2) were exported and differential expression analysis of individual proteins was performed using PEAKSviz (https://jdhogan.shinyapps.io/peaksviz/). To correct for multiple comparisons, false-discovery rate (FDR) was used in lieu of p-values. FDR < 0.05 is regarded as statistically significant. To examine affected protein groups, gene set enrichment analysis was performed using WebGestalt (Liao et al., 2019). To examine the relative levels of collagens, the total abundance of all collagens was found for each sample, and the contribution of individual collagens is given as a percentage of total collagen abundance. Student T-tests were performed to assess the differences between AD and control groups and a Bonferroni correction was applied to correct for multiple comparisons.

## Results

**Figure 1:**
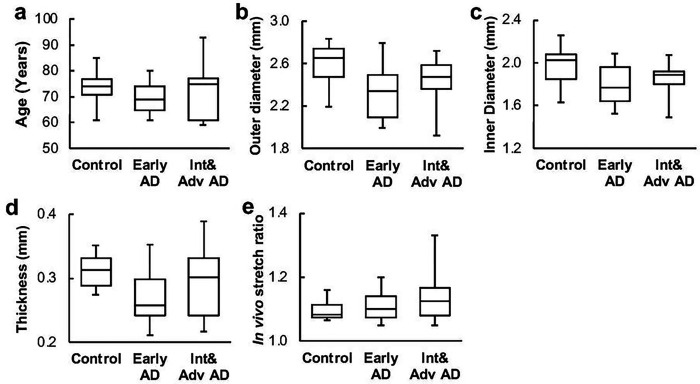
(a) Ages of the subjects from the control, early AD, and intermediate& advanced AD groups; undeformed ACA sample dimensions including (b) outer diameter, (c) inner diameter, and (c) thickness; and (e) *in vivo* stretch ratio of ACA samples.

The average age for each group was 72 ± 4.9, 70 ± 4.1, and 74 ± 3.3 for control, early AD, and intermediate & advanced stages, respectively, with no significant difference among the groups (Fig. 1 a). The average outer diameter, inner diameter, and thickness of ACAs from the AD groups decreased slightly compared to the control group (Table 1). The average *in vivo* axial stretch ratio also showed a slight trend of increase from 1.10 ± 0.01 to 1.11 ± 0.02 and 1.13 ± 0.02 as AD neuropathological change progressed for the control, early AD, and intermediate & advanced AD groups, respectively (Figs. 1 b–e). However, there was no significant difference among all groups in ACA dimensions and *in vivo* axial stretch ratio.

**Figure 2:**
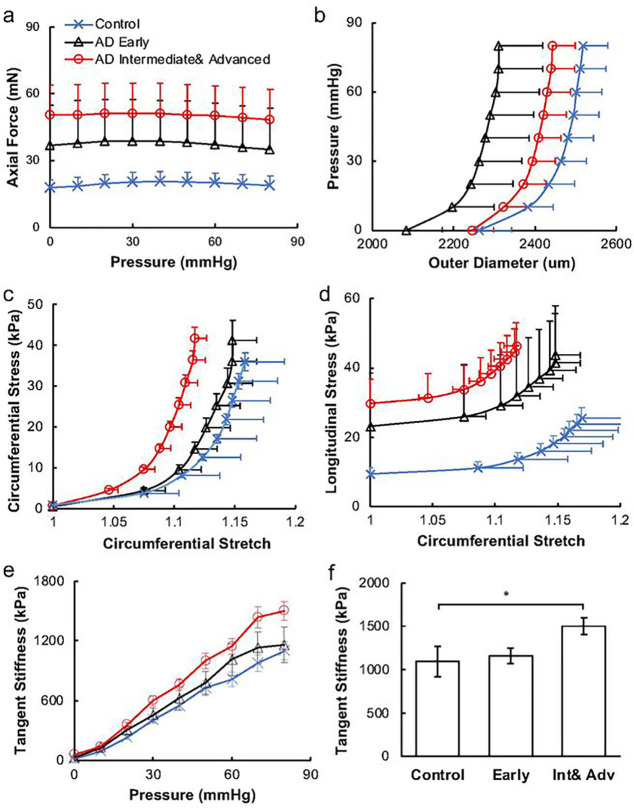
Mechanical response of ACAs from biaxial extension-inflation test at *in vivo* stretch ratio. (a) Axial force-pressure and (b) pressure-outer diameter measurements; (c, d) circumferential and longitudinal stress vs. circumferential stretch ratio calculated using Equation (3). (e) Tangent stiffness obtained from the circumferential stress vs. stretch curves in Figure 2c for the control, early AD, and intermediate & advanced AD groups. (f) Tangent stiffness at 80 mmHg among the three groups presented in Figure 2e. (*p<0.05)

Changes in the mechanical properties of ACAs with AD progression were studied using biaxial extension-inflation test (Fig. 2). At 80 mm Hg, the axial force increased significantly from 18.78 ± 4.40 mN for the control, to 45.93 ± 18.75 mN and 51.18 ± 13.68 mN for the early and intermediate & advanced AD groups, respectively (p < 0.05). The pressure-diameter response showed a leftward shift with AD progression. Consistently, as AD developed, the initial low stiffness region of the circumferential stress-stretch curve shortened (Fig. 2c). This was also manifested by the leftward shift of the maximum circumferential stress-stretch curves indicating artery stiffening with AD progression. The longitudinal stress-stretch curves also demonstrated a similar arterial stiffening behavior with shortened toe region and pronounced stress elevation in the intermediate & advanced AD group. To better analyze the arterial mechanical changes, the stress-stretch curves in the circumferential direction were fitted with exponential functions and derived to obtain tangent stiffness (Fig. 2e,f). When early AD developed, the tangent stiffness of stress-stretch curves in circumferential direction increased. The slope of advanced AD group and the intermediate& advanced AD group are significantly higher than that of control curve at 80mmHg (p < 0.05).

**Figure 3:**
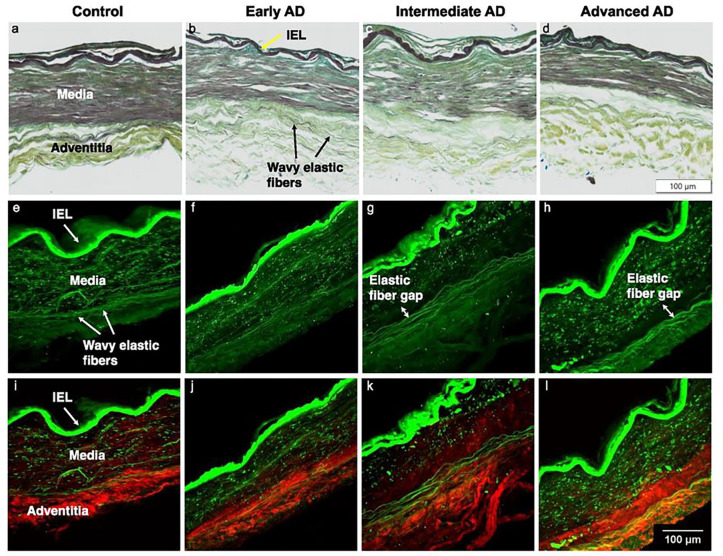
Representative histological and multiphoton images at the circumferential cross section of ACAs showing progressive cellular and extracellular structural changes with AD progression. (a, e, i) 72-year-old male (control), (b, f, j) 72-year-old male (Braak II to III, early AD), (c, g, k) 69-year-old male (Braak IV, intermediate AD), and (d, h, l) 79-year-old male (advanced AD). (a-d) Histological images with Movat’s stain (nuclei and elastin black, GAGs blue, and collagen yellow). (e-l) Multiphoton images with single elastin channel (e-h) and combined collagen and elastin channels (i-l) with collagen fibers in red and elastic fibers in green. The intimal layer of the ACAs faces upward in all images.

**Figure 4:**
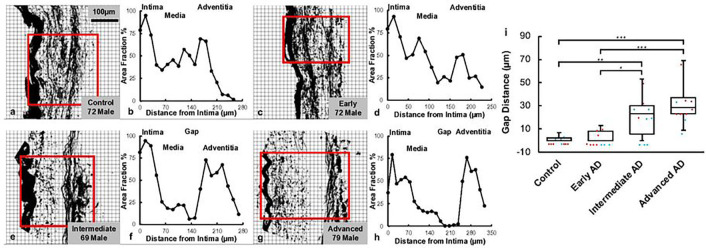
Quantification of gap distance within the elastic fiber network at the media-adventitia interface. (a, c, e, g) Binarized multiphoton images of elastic fibers from Figures 3e–3h with region of interest selected. (b,d,f,h) The corresponding area fraction occupied by black pixels was plotted with the intima, media, and adventitia regions indicated. (i) The gap distance in the elastic fiber network at the media-adventitia interface was quantified and plotted for the control, early AD, intermediate AD, and advanced AD groups obtained from multiphoton image quantification. The top and bottom of the box presents the first and third quartile of the data set. The extended bars from top and the bottom of the box represent the maximum and minimum. The horizontal line in the box represents the median. Red symbols represent data points from male, and blue symbols represent data points from female samples. (*p < 0.05, **p < 0.01, ***p< 0.001)

Microstructural study of ACA revealed a trend of media atrophy and adventitial layer disorganization with AD progression. Histological images with Movat’s stain showed a well-developed internal elastic lamella presented between the medial and the intimal layers (Figs. 3a–d). The media consists of SMCs, sparse elastic fibers, and small amounts of proteoglycan and collagen fibers. Collagen was abundant in the adventitial layer observed in the form of wavy bundles (Fig. 3a). Media atrophy was observed, characterized by the loss of SMCs and elastic fibers starting from the media-adventitia interface (Fig. 3b). When AD progressed, the atrophy region extended towards the lumen, causing a wider empty band with loosened structure, especially in the advanced AD stage (Figs. 3c and 3d). The progressive degradation of elastic fibers was further observed in multiphoton images taken at the circumferential cross section of ACA (Figs. 3e–l). Although the distribution of elastic fibers in the media was sparse, the loss of elastic fibers become evident with an empty band appearing at the media-adventitia interface at the intermediate & advanced AD stages (Figs. 3g, 3h, 3k, 3l). This empty band separated the elastic fiber network from the media and adventitia and was bordered by a thin layer of wavy elastic fiber at the interface of media and adventitia which did not seem to degrade as AD progresses. Collagen fibers in the media seemed to maintain a continuous and wavy configuration among all groups. The width of the empty band was quantified for all groups (Fig. 4). The average area fraction was 31.8 ± 8.5% in media and 47.8 ± 16.4% in adventitia. Based on this result, when an observable empty band appeared in the multiphoton image, the width of the band was determined when the area fraction value was below 14.8%. The width of the empty band in the elastic fiber network significantly increased in the intermediate and advanced AD groups (Fig. 4i). The average empty band width increases to 21.0 ± 15.4 μm in the intermediate and to and 32.8 ± 9.24 μm in the advanced AD group, while the empty band was unnoticeable in most of the control and early AD samples.

**Figure 5:**
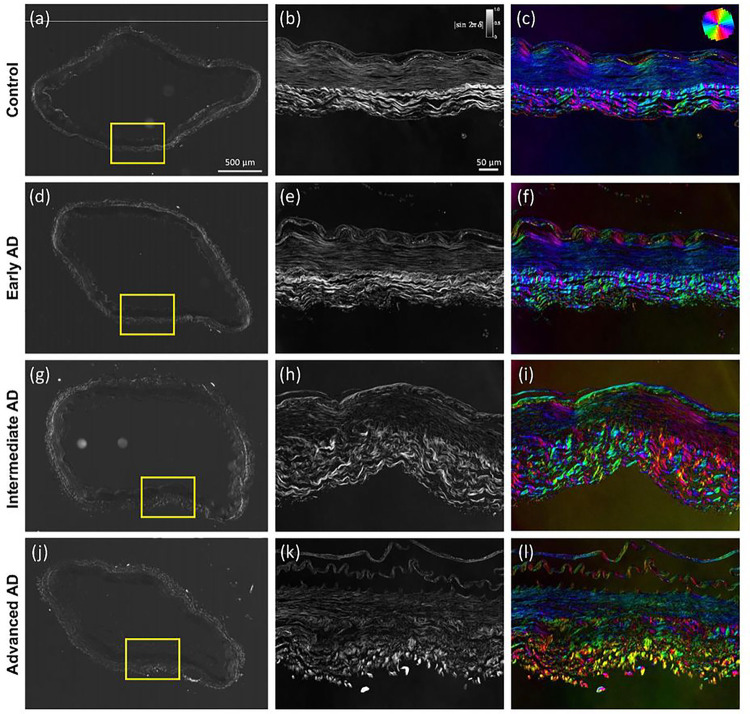
Representative structural birefringence imaged with qBRM of ACAs from Figure 3. (a,d,g,j) CCP-BRM was used to acquire the full-sample images of ACA cross section. qBRM was used to acquire the relative retardance images (b,e,h,k) and the retardance-weighted optic-axis orientation maps (c,f,i,l). The relative retardance maps are displayed based on the scale shown in the top-right corner of (b). The retardance-weighted optic-axis orientation maps are displayed based on the color orientation wheel in the top-right corner of (c) and the intensity of the relative retardance map.

The progressive changes in structural organization can be seen in the qBRM images presented in Fig. 5 For each sample, qualitative images of the entire section (Figs. 5a, 5d, 5g, 5j), were acquired with CCP-BRM. From each full-sample image, a region was selected for qBRM. In the relative retardance maps (Figs. 5b, 5e, 5h, 5k), the intensity of the media dropped off heavily with advanced AD as the extend of separation and loss of structural integrity of the SMC layer increased. The same general trend was observed in the relative retardance values of the adventitia as the extent of collagen fiber degeneration increased in advanced AD and the extent of alignment decreased. Furthermore, these structural changes were also represented in the optic-axis orientation maps (Figs. 5c, 5f, 5i, 5l), where the loss of structural integrity of the layers of the arterial wall resulted in a loss of its compact structure and uniform orientation. The qBRM images of the AD cases, specifically for collagen of the adventitia, showed a clear trend of increased disorder for each step further in AD progression. Collagen fibers were seen to shift from being more circumferentially aligned and wavy (Figs. 5c, 5f) to more disordered and separated as AD progresses (Figs. 5i, 5l). The qBRM images also showed evidence of structural breakdown and delamination, especially of the endothelial intimal layer and the internal elastic lamella.

**Figure 6:**
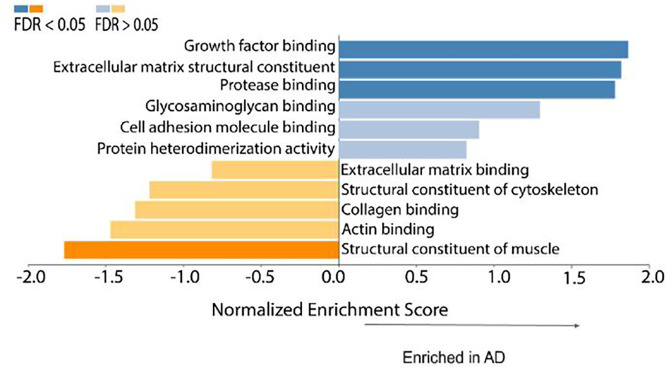
Gene set enrichment analysis based on proteomics data of ACAs from the control and advanced AD groups.

**Figure 7:**
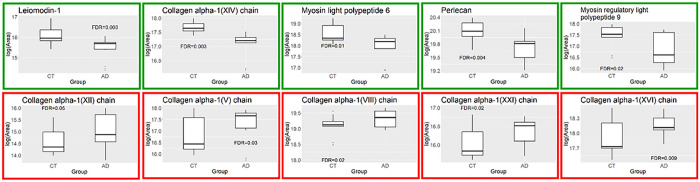
Differentially expressed proteins in the ACAs from the control and advanced AD groups. Plots in the top row show decreased protein abundance in AD, while plots in the bottom row show increased protein abundance in AD.

Mass spectrometry results showed that the relative amounts of collagen are altered in AD compared to controls (Table 2). The levels of type I collagen were increased in AD, and this increase was accompanied by a decrease in type IV collagen. Further, gene set enrichment analysis revealed that several groups of genes were significantly differentially expressed (FDR < 0.05) (Fig. 6). Growth factor binding (GO:0019838), ECM structural constituent (GO: 0005201) and protease binding (GO: 0002020) were significantly enriched in AD. These gene sets include type I collagen, HtrA serine peptidase 1, elastin, biglycan, and periostin. The structural constituent of muscle gene set (GO:0008307) was significantly under-expressed in AD. On an individual protein level, the abundances of several members of the collagen family were significantly increased in AD: types V, VII, XII, XVI, and XXI (FDR < 0.05) (Fig. 7). The levels of several smooth muscle cell-associated proteins decreased in AD ACA relative to controls, specifically perlecan, myosin light polypeptide 6 and myosin regulatory light polypeptide 9, and leiomodin-1 (FDR< 0.05) (Fig. 7).

## Discussion

This study focused on the structural and functional changes of human ACAs with AD progression. Changes in wall structure were observed including media atrophy, elastic fiber degradation and adventitial collagen disorganization, all of which likely contributed to progressive arterial stiffening with AD development. Histological staining revealed media atrophy, characterized by the loss of SMCs and elastic fibers, in the ACAs with AD. The atrophy initiated at the media-adventitia interface and propagated towards the lumen as AD developed (Fig. 3). Multiphoton imaging provided further evidence of the degradation of elastic fibers accompanying the loss of SMCs (Fig. 4). Elastic fiber degradation appeared in early AD stage and increased in later AD stages. As a result of elastic fiber degradation, an empty band absent of SMCs and elastic fibers was observed at the media-adventitia interface that widens significantly in later AD stages (Figs. 3 and 4). Although the role of elastic fibers in cerebral arteries and its degradation with AD remains to be understood, it is important to note that the media-adventitia interface contains perivascular nerves (Bleys et al., 1996). Cerebral arteries are innervated extrinsically to control vascular contraction (Cipolla, 2009). As AD progresses, significant perivascular innervation loss has been reported (Bleys et al., 1996). Among limited studies on perivascular innervation degradation and corresponding artery structural changes, elastin downregulation was reported with perivascular innervation degradation in femoral artery and abdominal aorta (Dab et al., 2012). Mass decrease in both middle and posterior cerebral arteries after perivascular innervation removal, indicating loss of ECM components or SMC in large cerebral arteries, has also been reported (Bevan et al., 1983; Perry et al., 1998). The cross-sectional area of elastin in rat cerebral arterioles decreases after sympathetic denervation (Baumbach et al., 1989). Innervation loss may damage blood flow regulation (Hamel, 2006), which may in turn lead to inadequate driving forces in Aβ drainage, further worsen media atrophy (Daulatzai, 2017; Jack C, 2000; Wierenga et al., 2014). The media atrophy at the media-adventitia interface observed in this study (Figs. 3–4), is especially intriguing because soluble Aβ was reported to drain along basement membrane between SMCs in the media (Carare et al., 2008; Weller et al., 2008), and deposited Aβ was found replacing SMC in smaller cerebral arteries at the media-adventitia interface (Weller et al., 1998). With media atrophy and Aβ elevation in AD, vascular pulsation is weakened, leading to more insufficient Aβ drainage (Weller et al., 2008). Evidence has also shown that when Aβ in artery wall interacts with endothelium, oxidative stress increases which further contributes to Aβ increase, neurodegradation, and possible cognitive decline (Thomas et al., 1996; Varadarajan et al., 2000).

Adventitial collagen disorganization is another major structural change observed in this study (Fig. 5). Due to the structural anisotropy and organization of ACA, the arterial components exhibit optical birefringence. Namely, strong birefringence is seen in the collagen fibers of the adventitial layers, while the elastic fibers and SMCs that make up most of the media exhibit weaker birefringence (Wolman, 1975). Using polarized light microscopy, the birefringence of these arterial components can be reliably imaged in a label-free manner (Smith et al., 1981) that enables the determination of the relative retardance and local orientation of structural anisotropy of cells and ECM components for every pixel in the image. These imaging approaches have been used to characterize collagen realignment across arterial layers with varying transmural pressure (Finlay et al., 1995) and to study regions of cerebral arteries susceptible to aneurysm (Canham et al., 1999; Rowe et al., 2003). In our study, the quantitative metrics from qBRM provided insights into the disorganization or decompaction of the layer structures in vasculature. As a function of AD severity, we observed a loss of compaction and integrity of SMCs in the media, evidenced by reduced intensity, as well as degeneration of collagen structure in the adventitia, evidenced by broader and more disoriented (more colorful) layers of collagen (Fig. 5). These observations demonstrated progressive structural breakdown in cerebral arteries with pathological AD development.

The structural changes in SMCs and ECM resulted in compromised arterial integrity and the mechanical properties of ACAs in AD progression (Fig. 2). As a result of SMC atrophy, the proportion of vessel wall thickness occupied by the SMCs was reduced (Fig. 3), leading to compromised contractility of cerebral arteries (Hald et al., 2016). The increased systematic arterial stiffening that AD patients experience always accompanies higher level of dementia and cognitive impairment (Hanon et al., 2005; Rabkin & Jarvie, 2011; Singer et al., 2014). Among limited studies that relate the cerebral artery wall stiffness and dementia, Rivera-Rivera et al. (Rivera-Rivera et a., 2021) observed increased intercranial artery stiffness in AD patients from noninvasive transcranial pulse wave velocity measurements by MRI, which aligned with our findings (Fig. 2). Higher arterial stiffness results in compromised blood perfusion, and the blood pressure increases correspondingly (King, 2014). Such high pressure in cerebral arteries is further passed down to smaller intracranial vessels, which can cause microvascular lesion such as microhemorrhage and vascular rupture (Henskens et al., 2008). In addition, chronic hypoperfusion can be the onset of sporadic AD due to damage on brain metabolism and cognitive function caused by long term insufficient oxygen and nutrients supply (de la Torre, 2018). Furthermore, in AD patients brain lesions emerged with high arterial stiffness and white matter hyperintensity was reported to cause decline in cognition (Henskens et al., 2008). ACA supplies blood to the frontal lobe, where integrity changes of white matter mainly occurs when AD first develop (God et al., 2012).

Mass spectrometry measurements correlated with reduced SMC activity in AD. Using gene set enrichment analysis, the structural constituent of muscle gene set was shown to be negatively enriched in AD (Fig. 6). This gene set consists principally of myosin family members and its under-expression is consistent with SMC dysfunction. Loss of myosin VI, in particular, has been linked to alterations in synaptic structure (Osterweil et al., 2005). In addition to being present in the structural constituent of muscle gene set, SMC components are also significantly decreased on an individual protein level (Fig. 7). Two myosin components, myosin light chains and Leiomodin-1, were observed at significantly lower levels in the ACAs from the advanced AD group (Fig. 7), which may have implications for the reduced smooth muscle contraction. Leiomodin-1, the level of which decreased in AD, has been detected in the aorta and in vascular smooth muscles in the lung (Conley et al., 2001; Sahoo et al., 2016). The *LMOD1* gene has been identified as a risk locus in coronary artery disease; its expression has been found to be reduced in SMCs within atherosclerotic lesions, and its reduced expression is associated with decreased cell contraction (Nanda et al., 2018). Decreased expression of myosin heavy chains has been associated with reduced force generation of SMCs and reduced arterial contractility (von Kleeck et al., 2021).

Mass spectrometry results further indicate aberrant ECM structure and assembly in AD. Two major subfamilies of collagens are fibrillar collagens and fibril-associated collagens with interrupted triple helices (FACIT). Fibrillar collagens contain one triple helical domain and often provide structure to the ECM, while FACITs contain multiple triple helices and assist in conferring biological activities to collagens (Ricard-Blum, 2011). The ECM structural constituent gene set, which includes several fibrillar and FACIT collagens was positively enriched in AD, indicating that the structure and biological activity of the ECM is dysregulated in the AD cerebrovasculature (Fig. 6). A similar enrichment of the ECM structural constituent gene set has also been observed in Parkinson’s disease (Raghunathan et al., 2020; Downs et al., 2022). In the present study, the changes in the expression of collagen type genes are likely contribute to the changes of biomechanical function in ACAs.

The relative levels of individual collagens are also affected in the AD ACA (Table 2). The most pronounced changes to overall collagen composition are an increase in type I collagen in AD and a corresponding decrease in type IV collagen. Similar changes have been observed in small vessel disease, where an increase in fibrillar type I and III collagens is accompanied by a decrease in nonfibrillar type IV collagen (Kumar et al., 2020). The increase in fibrillar collagens correlates with arterial wall thickening, while the decrease in type IV collagen is associated with basement membrane dysfunction. The abundance of collagens is also affected in the AD ACA (Fig. 7). Type XXI collagen, the level of which increased in AD, is known to contribute to the ECM assembly of the vascular network (Chou and Li, 2002). While little is understood about the function and relevance of type XXI collagen under pathological conditions, it is thought to mediate protein-protein interactions between fibrillar collagens, serving as a molecular bridge in the ECM (Beretov et al., 2014). Type XIV collagen, the level of which decreased in AD, is known to regulate collagen fibril formation, a function that is particularly important in tissues of high mechanical demand (Tao et al., 2012). Its absence has been shown to compromise mechanical properties of tissues including skin, tendons, and the myocardium (Tao et al., 2012; Ansorge et al., 2009).

Response to injury and stress in the cerebrovasculature are also dysregulated in AD (Table 2, Fig. 7). Type V collagen, which is increased in AD, has been shown to interact with interleukins in the context of pulmonary arterial hypertension, thus pointing to a possible inflammatory role (Batah et al., 2020). Another FACIT collagen that is dysregulated is type XII, the expression of which is increased in AD and has been demonstrated to increase with shear stress (Jin et al., 2003). While little is known about its role in the cerebrovasculature, it has been identified as a promoter of axonal regeneration after spinal injury in a zebrafish model (Wehner et al., 2017). Also enriched in AD was type VIII collagen, which is an important component of the endothelium of blood vessels. Type VIII collagen is expressed by vascular SMCs, and its expression increases in response to vascular injury, particularly in the media and neointima; further, it has been shown to be upregulated in diseases associated with vascular remodeling and angiogenesis (Sibinga et al., 1997; Hansen et al., 2016). Along with other factors, collagens V, VIII, XIV, and XVI have been shown to be upregulated after cardiac injury, altering the mechanical properties of scar tissue (Yokota et al., 2016). While the exact roles for the dysregulation of these different types of collagens in the cerebrovasculature remains to be elucidated, our study points to their potential involvement in inflammation and response to vascular injury. AD is associated with inflammation; analyses of proteomic and glycoproteomic data have revealed that inflammation-related proteins, including histocompatibility complex proteins, are over-represented in AD relative to controls (Seyfried et al., 2017; Zhang et al., 2020). Further study is needed to determine whether these and other collagens have a role in mediating inflammation and injury response in the cerebrovasculature.

### Limitation

The study was limited to analysis of the ACA. However, the middle cerebral artery as well as downstream arterioles may be more relevant for the development of early medial temporal lobe pathology that occurs in AD. Future studies should examine more regions and larger numbers to separately examine associations with beta-amyloid and tau pathology. There was no consideration of sex-dependency in AD development, which deserves further investigation. Arteries were inflated to mean arterial pressure (80 mmHg). Future studies considering the physiological pressure range may add information on the stress-stretch behavior within a broader pressure range. Arteries from tissue bank were kept frozen. Future studies could use freshly procured tissue to account for contributions from the SMCs to the mechanical response. Structural changes in the arterial wall can be location specific, however imaging, and structural analysis was limited to a few regions due to its relatively small field of view. Future studies can expand on the imaging domain for a more comprehensive understanding of the arterial wall structures. To quantitatively evaluate the relationship between AD and structural breakdown of vasculature using qBRM, it will be necessary to investigate a larger number of arterial sections from more patients with various stages of AD. For these future investigations, it will be important to ensure that arterial structure is optimally preserved during fixation and slide preparation, as quantifying structural disorganization with metrics will require consistent and reliable tissue sections for imaging. The proteomics study was performed using data-dependent acquisition, which is biased toward abundant species. Thus, information about less-abundant proteins may be lost. For example, elastin is a key player in the maintenance of arterial mechanics, but its sequence coverage is low in this study. Additionally, to better understand the correlation between mechanical changes and proteomic changes, analysis of individual layers of the arterial wall may be warranted.

## Conclusion

This study integrates advanced optical imaging, mechanical characterization, and mass spectrometry to reveal the structural and mechanical changes in ACAs with AD progression. Progressive media atrophy, elastic fiber degradation, and adventitia structural disorganization were observed with AD development. Results from biaxial inflation extension tests further confirmed the gradual arterial stiffening as AD progresses. Dysregulation in the SMC and ECM gene sets points to reduced SMC activity, aberrant ECM assembly, and the potential inflammatory response to vascular injury in AD cerebrovasculature. Future studies are underway to understand how these cerebrovascular changes correlate with pathological changes in the brain and with AD progression.

## Figures and Tables

**Table 1:** List of samples with donor age, gender, Braak stages, and measured arterial dimensions. Average values were presented as mean ± standard deviation.

Group	Age	Gender	Stage	Thickness (mm)	Inner diameter (mm)	Outer diameter (mm)
*Alzheimer’s disease*						
**Early**	66	Male	Early	0.35	2.09	2.79
				0.25	2.00	2.50
	61	Female	Early	0.21	1.63	2.05
				0.27	1.69	2.22
	80	Female	Braak II to III	0.24	1.52	2.00
	72	Male	Braak II to III	0.31	1.85	2.46
**Average**	69.8 ± 8.2			0.27 ± 0.05	1.80 ± 0.22	2.34 ± 0.30
**Intermediate**	77	Female	Braak III to IV	0.22	1.49	1.92
	79	Female		0.32	2.07	2.72
	69	Male	Braak IV	0.33	1.87	2.54
				0.34	1.78	2.47
**Average**	75.0 ± 5.3			0.30 ± 0.06	1.80 ± 0.24	2.41 ± 0.34
**Advanced**	63	Male	Braak V	0.35	1.89	2.59
	78	Female	Braak V	0.24	1.88	2.35
				0.22	1.49	1.93
	61	Male	Braak V	0.28	1.92	2.48
				0.24	1.87	2.35
	62	Male	Braak VI	0.25	1.9	2.40
	63	Female	Advanced	0.31	1.77	2.38
	95	Female	Advanced	0.39	1.90	2.68
	83	Male	Braak V	0.32	2.07	2.7
	79	Male	Advanced	0.30	1.98	2.57
**Average**	73.0 ± 12.6			0.29 ± 0.05	1.87 ± 0.15	2.44 ± 0.22
**Control**						
	59	Female	-	0.27	1.85	2.40
				0.33	1.83	2.50
	72	Female	-	0.28	1.63	2.20
				0.29	2.26	2.84
	83	Male	-	0.35	2.07	2.78
				0.33	2.07	2.70
	72	Male	-	0.31	2.00	2.61
				0.31	2.11	2.73
**Average**	71.5 ± 9.8			0.31 ± 0.03	1.98 ± 0.20	2.60 ± 0.22

**Table 2:** Relative abundances of each quantified collagen type in the control and advanced AD groups. Values were presented as mean ± standard deviation for each group.

Collagen Type	Control (%)	AD (%)
**I**	43.52 ± 16.42	54.14 ± 17.38
**II**	0.26 ± 0.08	0.33 ± 0.17
**III**	26.98 ± 4.43	24.10 ± 4.35
**IV**	23.11 ± 10.47	16.32 ± 15.59
**V**	0.07 ± 0.01	0.10 ± 0.03*
**VI**	4.52 ± 1.77	3.51 ± 3.02
**VIII**	0.76 ± 0.30	0.81 ± 0.58
**XII**	0.01 ± 0.00	0.02 ± 0.02
**XIV**	0.20 ± 0.10	0.10 ± 0.09
**XVI**	0.22 ± 0.05	0.24 ± 0.15
**XVIII**	0.30 ± 0.14	0.30 ± 0.26
**XXI**	0.03 ± 0.01	0.04 ± 0.03

* p < 0.05

## Data Availability

Materials described in the manuscript will be available upon contacting the contact author.
